# Intensity-Modulated Radiation Therapy for Bilateral Choroidal Metastases Involving Macula and Optic Disc

**DOI:** 10.7759/cureus.46729

**Published:** 2023-10-09

**Authors:** Kenya Kubo, Noriyasu Hashida, Atsushi Watanabe, Kazuichi Maruyama, Ryoong-Jin Oh, Kohji Nishida

**Affiliations:** 1 Department of Ophthalmology, Osaka Rosai Hospital, Sakai, JPN; 2 Department of Ophthalmology, Osaka University Graduate School of Medicine, Osaka, JPN; 3 Integrated Frontier Research for Medical Science Division, Institute for Open and Transdisciplinary Research Initiatives (OTRI), Osaka University Graduate School of Medicine, Osaka, JPN; 4 Department of Radiation Oncology, Miyakojima IGRT (Image Guided Radiation Therapy) Clinic, Osaka, JPN

**Keywords:** radiation retinopathy, radiation-induced optic neuropathy, breast cancer, intensity-modulated radiation therapy (imrt), metastatic choroidal tumor

## Abstract

This case report discusses the case of a 76-year-old woman with choroidal metastasis from breast cancer who was treated with intensity-modulated radiation therapy (IMRT). Choroidal metastasis is a common ocular tumor, and the occurrence of this condition has increased due to improved diagnostic tools and longer survival of metastatic patients. IMRT is an innovative radiation therapy technique that reduces complications and improves the curative effect by concentrating radiation on the tumor while minimizing exposure to surrounding tissues. In this case, the patient had a history of breast cancer and was undergoing chemotherapy when she presented with vision loss and blurred vision. Imaging tests confirmed choroidal metastasis, and IMRT was performed under the guidance of a radiation oncologist. After treatment, the choroidal lesion dramatically reduced in size, and the patient's vision improved. The text concludes that radiation therapy, including IMRT, is becoming more common as a treatment for ocular metastasis to improve vision and preserve the eye. When choosing radiation therapy, it is essential to consider the size of the tumor and the impact on surrounding tissues. IMRT is an effective treatment that enables precise and concentrated irradiation of the tumor tissue while minimizing exposure to normal tissues.

## Introduction

Metastatic choroidal tumors are the most common intraocular choroidal lesions, with an increasing frequency owing to the long survival of metastatic patients and improvements in diagnostic tools [1-3]. Breast cancer is the most frequent primary tumor site for choroidal metastases, followed by lung cancer as the second most common site [2-5]. The optimal treatment for these patients is controversial, and it is important to consider their life expectancy when considering the treatment. Most patients with cancer have a restricted life span and require easily implementable and efficient treatment. However, recent advancement in anti-cancer therapies has led to an elevated likelihood of survival for certain individuals. Effective and long-lasting treatment can be beneficial for such patients [6]. Thariat et al. proposed an algorithm for selecting treatment options for choroidal metastases [7]. For patients with a limited life expectancy, it is recommended that systemic therapy be prioritized. Short hypofractionated external beam radiation therapy (EBRT) may be considered if systemic therapy is inadequate. In patients with a prolonged life expectancy, it is recommended to consider EBRT alongside systemic therapy.

The most commonly used treatment for metastatic choroidal tumors is EBRT [6]. However, there are few reports on the specific planning methods for treatment with EBRT. Intensity-modulated radiation therapy (IMRT) is a modern radiation technique that has evolved from EBRT [8]. IMRT uses multiple fields, typically five to nine, and modulates the radiation dose distribution using a multileaf collimator. The results are complex concave dose distributions providing greater precision and better conformality to the tumor volume than conventional EBRT by reducing the radiation to the surrounding tissues and targeting only the tumor [8,9]. We report a case of multiple metastatic choroidal tumor lesions located near the macula and optic disc in a breast cancer patient treated with IMRT.

## Case presentation

**Figure 1:**
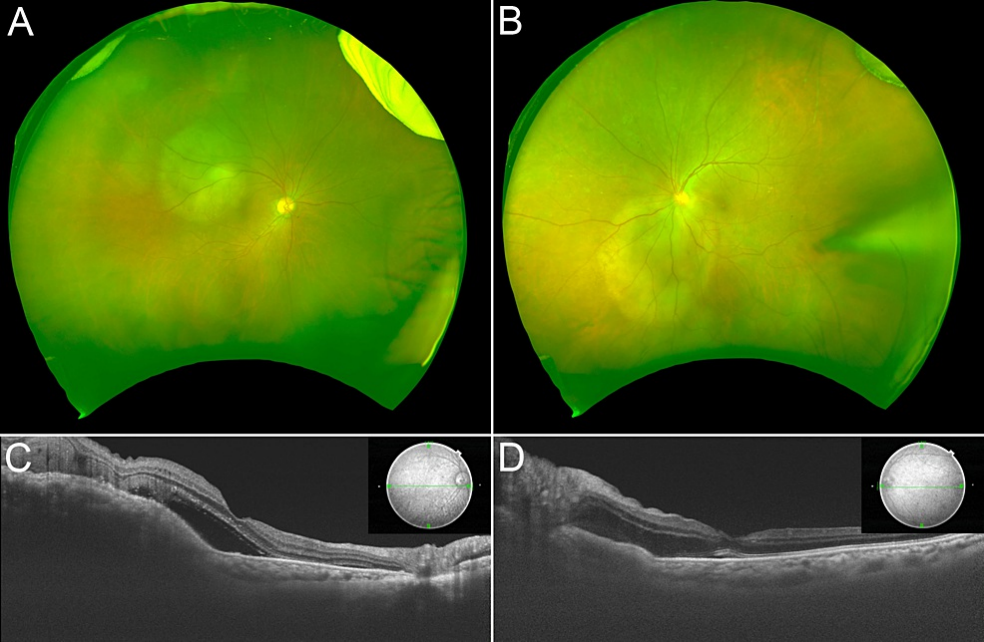
Ophthalmologic findings at the first visit Ultra-widefield (UWF) fundus images exhibiting the tumors involved in the macula in the right eye (A) and the optic disc in the left eye (B). Optical coherence tomography (OCT) findings before intensity-modulated radiation therapy demonstrated elevated choroidal lesions and serous retinal detachment in the right eye (C) and disc elevation in the left eye (D).

The patient is a 76-year-old woman with cancer of the right breast. She underwent a total mastectomy followed by hormonal therapy with tamoxifen for five years. Eleven years after surgery, a recurrent lesion was found in the right supraclavicular lymph node, and surgical resection was attempted but was not completely successful. Letrozole was started, and the condition remained stable. After eight years, she visited a local ophthalmologist because of blurred vision. Slit-lamp examination revealed mild cataracts in both eyes, fundus examination showed a choroidal lesion in the right eye, and optical coherence tomography (OCT) showed subretinal fluid (SRF) and subretinal detachment in the right eye. Two months later, a choroidal lesion appeared in the left eye. The patient was referred to our clinic. Corrected visual acuity was 0.5 in both eyes. Ultra-widefield (UWF) fundus photography and OCT revealed an elevated choroidal lesion near the macula in the right eye and the optic disc in the left eye along with serous retinal detachment (Figures 1A-1D).

**Figure 2:**
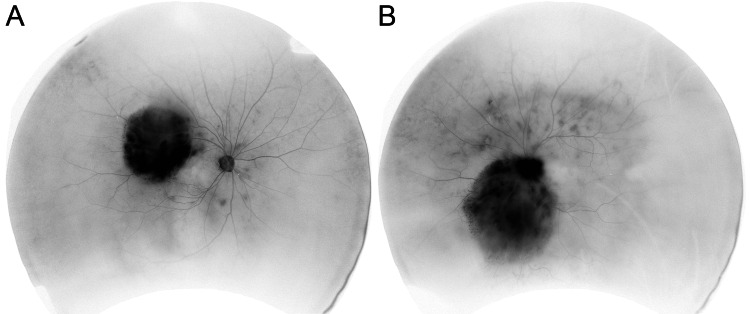
Fluorescein angiography Black-and-white reverse imaging of fluorescein angiography showing the infiltration of the main lesion involved in the macula in the right eye (A) and the optic disc in the left eye (B), and micrometastases.

Considering the patient's breast cancer history, metastases throughout the body, and ophthalmologic imaging findings, a diagnosis of a metastatic choroidal tumor was made. Fluorescein angiography (FA) revealed the infiltration of the main lesion and micrometastases (Figures 2A, 2B).

**Figure 3:**
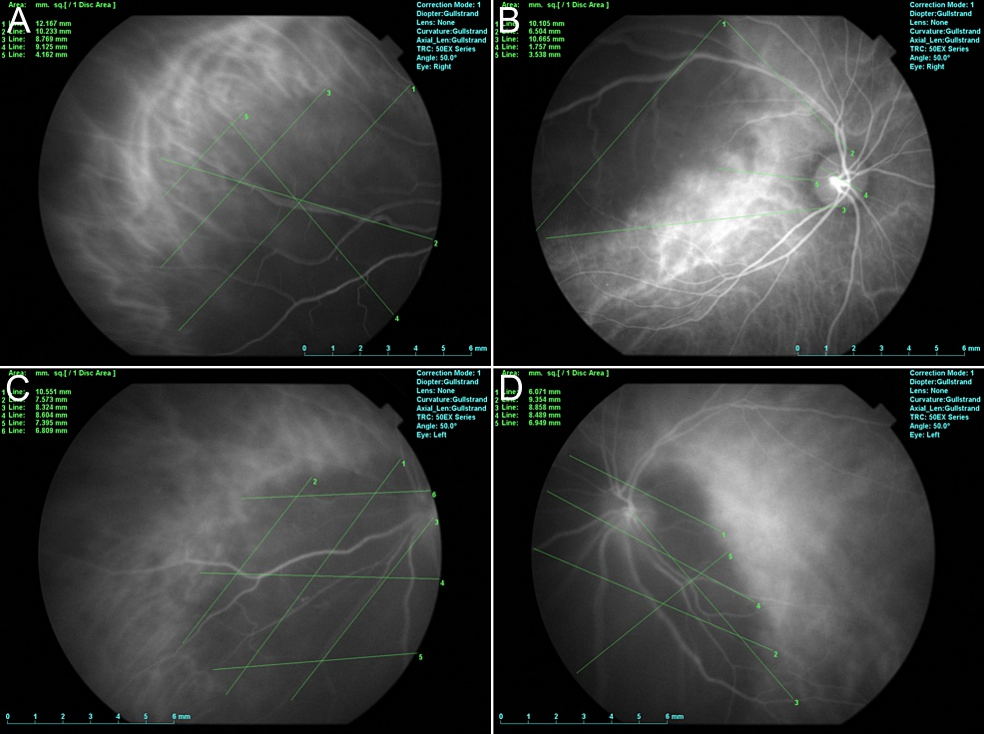
Indocyanine green angiography The actual tumor size in the choroid is measured using indocyanine green angiography during radiation therapy (A-D).

Indocyanine green angiography (IA) revealed the spread of the main metastatic hypofluorescent lesion (Figures 3A-3D).

**Figure 4:**
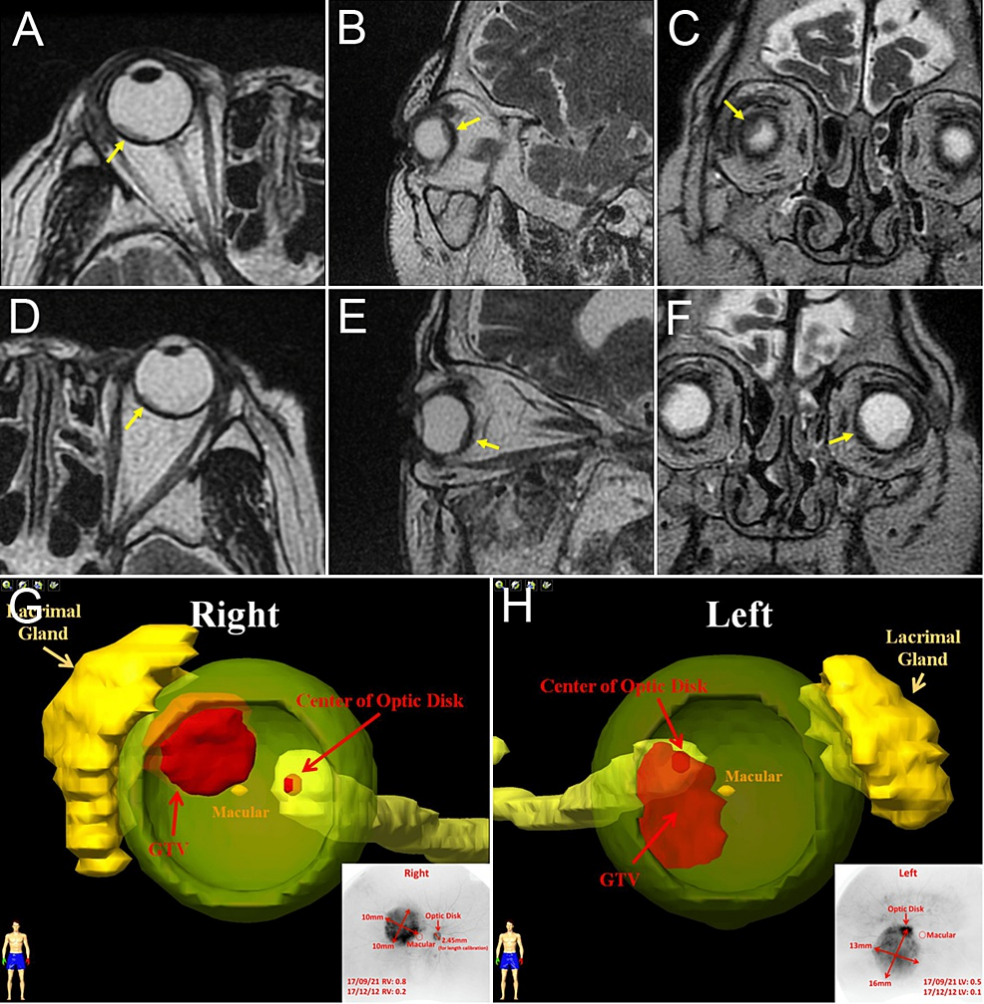
IMRT planning for the bilateral metastatic choroidal tumors Axial, sagittal, and coronal T2-weighted magnetic resonance imaging (MRI) slices of the right eye (A-C) and left eye (D-E) showing the main choroidal lesions (arrows). The extent of radiation exposure is determined by integrating the findings from fundus angiography and MRI and creating three-dimensional models of the tumors (G, H). IMRT: intensity-modulated radiation therapy, GTV: gross tumor volume.

The radiation field for the choroidal lesion was determined by superimposing ophthalmic imaging information on the magnetic resonance imaging findings (MRI) (Figures 4A-4H).

**Figure 5:**
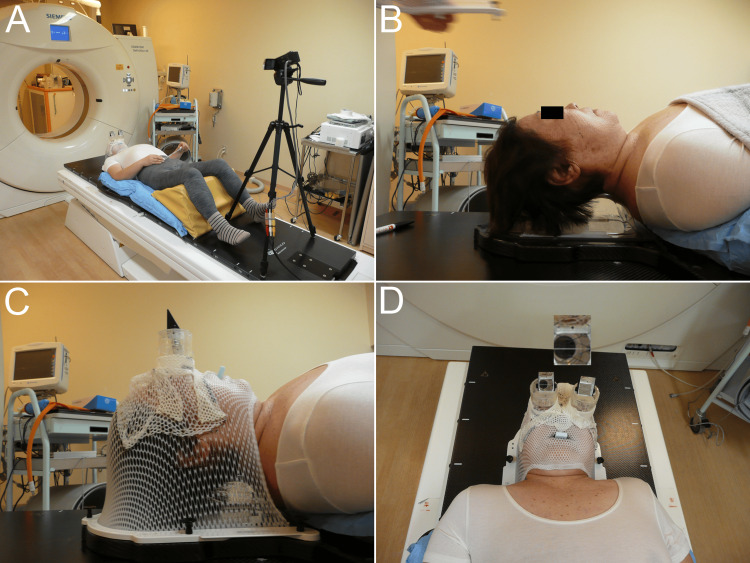
The fixation and continuous monitoring device The device consists of an immobilization shell, a right-angle prism mirror, a charge-coupled device (CCD) camera, and a guide lamp (A). The patient lying on the bed in the supine position (B) and the head was immobilized (C). Eye movements and fixations were continuously monitored using a CCD camera (D).

Static IMRT was performed under the guidance of a radio oncologist. Eye fixation and monitoring during irradiation were performed using a right-angle prism mirror as previous report [10]. Non-coplanar IMRT was considered to be the appropriate treatment because it allows for higher-dose concentration to be delivered to the tumor while reducing the dose to surrounding organs at risk (OAR). This is particularly important for cases with bilateral choroidal metastases where the dose to the contralateral lesion would be higher with coplanar IMRT. It is also recommended to avoid the mirror part of the fixture when using an ocular monitoring system (Figures 5A-5D).

Dose plan metrics showing OAR received dose for planning target volume (PTV) and boost volume is shown in Tables 1, 2.

**Table 1:** Dose plan metrics for total choroidal irradiation Rt.: right, Lt.: left, vol.: volume.

Eyeball		Retina		Optic Nerve		Macular		Optic Disk		Lacrimal Gland	
Rt. eyeball (vol. 6.44cc)	Lt. eyeball (vol. 7.27cc)	Rt. retina (vol. 2.34cc)	Lt. retina (vol. 2.67cc)	Rt. optic nerve (vol. 0.76cc)	Lt. optic nerve (vol. 0.58cc)	Rt. macular	Lt. macular	Rt. optic disk	Lt. optic disk	Rt. lacrimal gland (vol. 1.40cc)	Lt. lacrimal gland (vol. 1.00cc)
Max dose: 110%, Mean dose: 95.1%, D0.5cc: 104.8%, D1cc: 104.1%, D5cc: 93.3%	Max dose: 110%, Mean dose: 98.5%, D0.5cc: 106.8%, D1cc: 106.1%, D5cc: 100.5%	Max dose: 111%, Mean dose: 103.6%, D0.1cc: 106.9%, D0.5cc: 104.7%, D1cc: 103.9%, A100%: 1154mm^2^, A90%: 1171mm^2^, A80%: 1171mm^2^, A70%: 1171mm^2^	Max dose: 110%, Mean dose: 104.7%, D0.1cc: 107.8%, D0.5cc: 106.2%, D1cc: 105.4%, A100%: 1316mm^2^, A90%: 1337mm^2^, A80%: 1337mm^2^, A70%: 1337mm^2^	Max dose: 104%, Mean dose: 22.3%, D0.1cc: 59.2%, D0.5cc: 1.63%	Max dose: 102%, Mean dose: 28.6%, D0.1cc: 54.6%, D0.5cc: 7.4%	Point dose: 102.7%	Point dose: 104.9%	Point dose: 103.8%	Point dose: 105.3%	Max dose: 104%, Mean dose: 78.1%, D0.1cc: 99.0%, D0.5cc: 86.7%, D1cc: 68.5%	Max dose: 107%, Mean dose: 78.2%, D0.1cc: 100.9%, D0.5cc: 80.1%

**Table 2:** Dose plan metrics for boost irradiation of ocular tumors Rt.: right, Lt.: left, vol.: volume.

Eyeball		Retina		Optic Nerve		Macular		Optic Disk		Lacrimal Gland	
Rt. eyeball (vol. 6.39cc)	Lt. eyeball (vol. 7.34cc)	Rt. retina (vol. 2.36cc)	Lt. retina (vol. 2.80cc)	Rt. optic nerve (vol. 0.65cc)	Lt. optic nerve (vol. 0.59cc)	Rt. macular	Lt. macular	Rt. optic disk	Lt. optic disk	Rt. lacrimal gland (vol. 0.75cc)	Lt. lacrimal gland (vol. 0.84cc)
Max dose: 107%, Mean dose: 36.3%, D0.5cc: 102.4%, D1cc: 86.6%, D5cc: 5.5%	Max dose: 110%, Mean dose: 53.4%, D0.5cc: 101.8%, D1cc: 96.6%, D5cc: 36.5%	Max dose: 107%, Mean dose: 46.4%, D0.1cc: 104.7%, D0.5cc: 93.9%, D1cc: 46.9%, A100%: 206mm^2^, A90%: 274mm^2^, A80%: 328mm^2^, A70%: 365mm^2^	Max dose: 108%, Mean dose: 59.0%, D0.1cc: 104.8%, D0.5cc: 101.3%, D1cc: 70.2%, A100%: 275mm^2^, A90%: 349mm^2^, A80%: 427mm^2^, A70%: 501mm^2^	Max dose: 96%, Mean dose: 18.8%, D0.1cc: 47.6%, D0.5cc: 0.64%	Max dose: 107%, Mean dose: 33.3%, D0.1cc: 94.4%, D0.5cc: 0.87%	Point dose: 103.0%	Point dose: 101.5%	Point dose: 78.7%	Point dose: 102.5%	Max dose: 106%, Mean dose: 79.7%, D0.1cc: 100.2%, D0.5cc: 72.1%	Max dose: 33%, Mean dose: 16.2%, D0.1cc: 22.5%, D0.5cc: 14.4%

**Figure 6:**
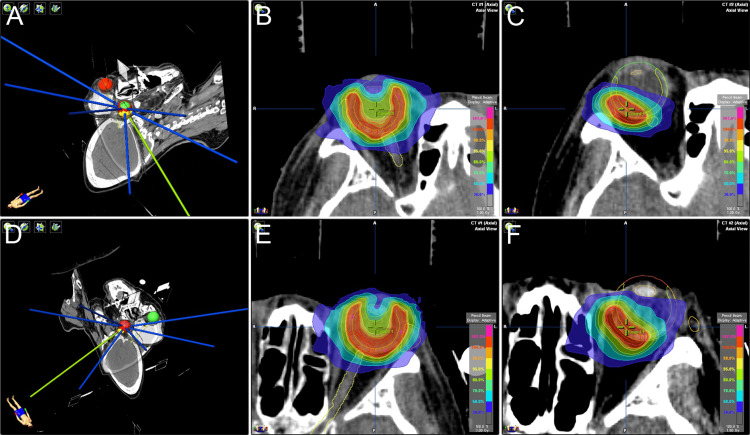
Practical application of radiation therapy for metastatic choroidal tumors IMRT is performed with the patient in the supine position and the head immobilized (A, D). The entire choroid in both eyes is irradiated, targeting the main lesions and micrometastases identified on fluorescein and indocyanine angiography (B, E). Boost irradiation is performed exclusively by focusing on the main lesions (C, F). IMRT: intensity-modulated radiation therapy.

**Figure 7:**
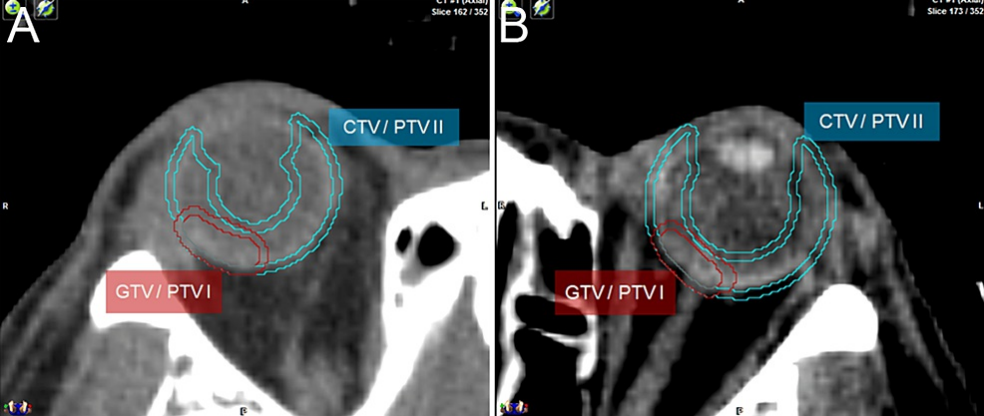
Axial slice of planning CT for choroidal tumor Axial slice of planning CT showing clinical target volume (CTV)/planning target volume (PTV) of 33Gy entire choroid irradiation and gross tumor volume (GTV)/PTV of 18Gy boost volume in the right (A) and left (B) eyes.

After irradiating the entire choroid with 33Gy (3.00Gy x 11 sessions: equivalent total doses (EQD2): 35.75Gy (α/β=10), 41.25Gy (α/β=2)) (Figures 6A, 6B, 6D, 6E) and boosting the main lesion with 18Gy (1.80Gy x 10 sessions: EQD2: 17.70Gy (α/β=10), 17.10Gy (α/β=2)) (Figures 6C, 6F, 7A, 7B), dramatic shrinkage of the choroidal lesion and prompt disappearance was observed.

**Figure 8:**
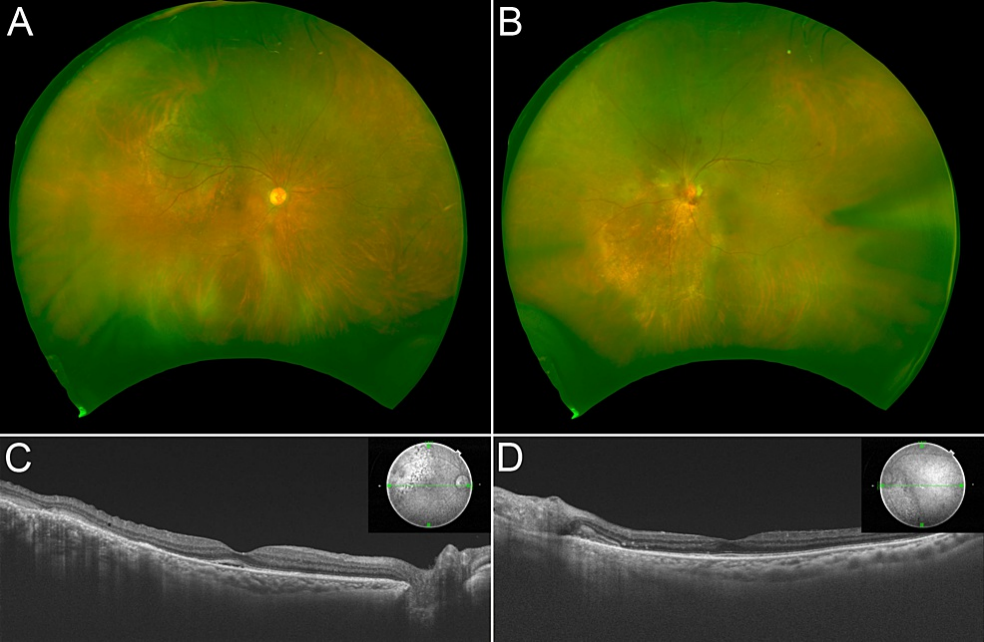
Ophthalmologic findings after IMRT UWF (A, B) and OCT (C, D) images after IMRT demonstrated an obvious loss of subretinal fluid and tumor regression in both eyes. IMRT: intensity-modulated radiation therapy, UWF: ultra-widefield, OCT: optical coherence tomography.

Choroidal lesions were evaluated monthly after radiotherapy using UWF fundus photography for wide-angle evaluation of tumor extension and OCT to assess the status of retinal choroidal elevation. Consequently, long-term lesion control was achieved (Figures 8A-8D). The patient died 12 months after radiation therapy, during the observation period. However, long-term lesion control and maintenance of visual acuity without recurrence were achieved.

## Discussion

The existing literature provides treatment options for choroidal metastases. EBRT is the most widely used treatment, however; few authors have reported the details of radiotherapy treatment planning and EBRT technique [6]. Conventional EBRT has the potential to subject OAR to radiation doses that exceed acceptable limits. Brachytherapy is a potential treatment option for choroidal metastases, but it may be impractical for tumors located near the macula and optic disc due to the need for suturing a radioactive plaque to deliver radiation. Recently, in patients with choroidal metastases, several IMRT techniques have been reported. IMRT is an effective treatment enabling the precise and intensive irradiation of tumor tissues by calculating the radiation dose [9]. By combining two-dimensional information obtained from multimodal ophthalmic imaging with three-dimensional information obtained from MRI, difficult-to-treat lesions near the macula and optic nerve head can be managed by controlling the irradiation area. IMRT, which combines imaging information from ophthalmology and radiology, may be an option for treating ocular metastases.

We treated choroidal lesions in the vicinity of the macula and optic nerve papilla, which are generally difficult to treat unless the irradiation range is strictly controlled using IMRT. When selecting radiation therapy, the size of the tumor, along with the control of metastatic lesions and the impact of irradiation on the surrounding normal tissues must be considered [11]. Among the structures at risk in the eye, radiation-induced injury to the optic nerve or retina causes vision loss; hence, the dose should be kept below 55Gy and 45Gy, respectively [7-9]. In this case, although the main lesions were adjacent to the macula and optic disc, 51Gy of radiation was delivered through IMRT, allowing the tumor to regress and preserving long-term vision. Moreover, IA is the optimal imaging modality for choroidal lesions as it provides direct visualization of the lesion. The precise localization and radiotherapy of tumors were made possible by utilizing IA and MRI images.

There are some limitations to this study. Firstly, the optimal dose for choroidal micrometastases is not known. Therefore, it is questionable whether 33Gy radiation to the entire choroid was appropriate. Monitoring the long-term progress of cancer patients with metastatic disease poses a challenge, making it difficult to evaluate the efficacy of radiotherapy within a 12-month timeframe. Furthermore, the location and number of metastatic sites and the origin of the tumor vary from case to case, making consistent assessment difficult. Despite these limitations, we have demonstrated the prolonged efficacy of IMRT in managing choroidal metastatic lesions.

## Conclusions

Metastatic choroidal tumors are a vision-threatening complication in cancer patients. In this report, we describe a case of metastatic choroidal tumors close to the macula and optic disc, which may deter us from radiation therapy due to the risk of radiation complications. IMRT combined with ophthalmic and radiological imaging information can be a treatment option for these types of tumors.
